# SKA3 Promotes tumor growth by regulating CDK2/P53 phosphorylation in hepatocellular carcinoma

**DOI:** 10.1038/s41419-019-2163-3

**Published:** 2019-12-05

**Authors:** Yuchen Hou, Ziming Wang, Shanzhou Huang, Chengjun Sun, Jingya Zhao, Jiayu Shi, Zhongqiu Li, Zekang Wang, Xiaoshun He, Nga Lei Tam, Linwei Wu

**Affiliations:** 1 Department of Organ Transplantation, The First Affiliated Hospital of Sun Yat-Sen University, Guangzhou, 510080 China; 2 Department of Liver Surgery, Ren Ji Hospital, School of Medicine, Shanghai Jiao Tong University, 1630 Dongfang Road, Shanghai, 200127 China; 3 Department of Biliary and Pancreatic Surgery, The First Affiliated Hospital of Sun Yat-sen University, Guangzhou, 510080 China; 4 Department of General Surgery, Guangdong Provincial People’s Hospital. Guangdong Academy of Medical Sciences, Guangzhou, 510030 China; 5 The First Affiliated Hospital of Jinan University, Guangzhou, 510630 China; 6 Guangdong Provincial Key Laboratory of Organ Donation and Transplant Immunology, The First Affiliated Hospital, Sun Yat-sen University, Guangzhou, China; 7 Guangdong Provincial International Cooperation Base of Science and Technology (Organ Transplantation), The First Affiliated Hospital, Sun Yat-sen University, Guangzhou, China; 8 Digestive Medical Center, The Seventh Affiliated Hospital of Sun Yat-sen University, Shenzhen, 518107 China

**Keywords:** Liver cancer, Prognostic markers

## Abstract

Spindle and kinetochore-related complex subunit 3 (SKA3) is a component of the spindle and kinetochore-related complexes and is essential for accurate timing of late mitosis. However, the relationship between SKA3 and hepatocellular carcinoma (HCC) has not yet been fully elucidated. Gene expression omnibus (GEO) (GSE62232, GSE45436, GSE6764, and GSE36376) and The Cancer Atlas (TCGA) datasets were analyzed to identify differential expression genes. Cell proliferation ability was analyzed using Cell Counting Kit-8 (CCK8) assay and plate clone formation assay, while scratch wound healing assay and transwell assay were used to analyze cell invasion. The role of SKA3 in vivo was explored using subcutaneous xenotransplantation model and lung metastasis model. Bioinformatics analysis found that hepatocellular carcinoma patients with high levels of expression of SKA3 have a poor prognosis. Similarly, immunohistochemical staining of 236 samples of tumors also found higher SKA3 expression in them, than in adjacent normal liver tissues. Significant levels of inhibition of in vivo and in vitro tumor proliferation and invasion result from the downregulation of SKA3. Mechanistically, SKA3 was found to affect tumor progression through the cell cycle and P53 signaling pathway as shown by the gene enrichment analysis (GSEA). G2/M phase arrest and severe apoptosis was also found to result from SKA3 knockdown, as shown by the inhibition of CDK2/p53 phosphorylation together with downregulation of BAX/Bcl-2 expression in HCC cells. Overall, these findings uncover the role of SKA3 in regulating the apoptosis and proliferation of hepatocellular carcinoma cells. This study was able to uncover new information on the tumorigenesis mechanism in hepatocellular carcinoma.

## Introduction

Hepatocellular carcinoma (HCC) related deaths account the second highest proportion of cancer-related death worldwide, and is the cause of over 90% of all deaths from primary liver cancer^1^. Over the past decades, its incidence has increased dramatically^2^. Most liver cancers found in western countries arise as a result of cirrhosis caused by viral hepatitis, alcoholism, metabolic syndrome, and other rare diseases^3^. However, only 30–40% of all HCC cases are diagnosed early enough to receive treatment, such as resection, liver transplantation (LT), and/or percutaneous ablation. LT is the best treatment for hepatocellular carcinoma in patients who are unsuitable for resection because it removes tumors and potentially cirrhosis^4–6^. Although systemic therapy has clinical benefits, the prognosis of patients is mild and gradual. Therefore, the new treatment of liver cancer is still an unmet medical need. Currently, important insights into the biology of the disease have been gained through genome, transcriptional, and epigenomic studies^7^. However, HCC mechanism has not yet been fully elucidated. Therefore, further studies on HCC progression may lead to the identification of new therapeutic targets.

Spindle and kinetochore-related complex subunit 3 (SKA3) is an integral component of the SKA complex which is required for accurate chromosome segregation in mitosis^8^. SKA complex works with the KMN (KNL-1, Mis12, and Ndc80) network and stabilizes the kinetochore–microtubule interface^9–11^. CDK1 phosphorylates SKA3 specifically during mitosis which is required for the kinetochore localization of the SKA complex^12^. Furthermore, SKA3 competes with BUB1/BUBR1 and integrate BUB3 through its GLBS motif. The combination of SKA3 and BUB3 will release BUB1 and BUBR1, thus helping to silence spindle-assembly checkpoint (SAC)^13–15^. Previous studies have shown that overexpression of SKA3 is closely related to occurrence and development of variety kinds of tumors^16–18^. However, the role of SKA3 in HCC remains unclear. In this study, for the first time, elevated SKA3 expression levels were detected in HCC and its specific mechanism of action was explored.

The p53 gene has been widely studied and has been identified as a tumor suppressor gene that plays a prominent role in cell cycle regulation, repairing damaged DNA, scavenging free radicals and regulating immune response^19–21^. Mutant p53 can act as a transcription factor, regulating downstream target gene expressions, which leads to tumorigenesis^22,23^. Mutant p53 can also bind with other anti-oncogenes of p53 family, such as p63 and p73, inhibiting their functions of promoting apoptosis and repairing damaged DNA, leading to malignant cell growth^24–26^. Cyclin-dependent kinase 2 (CDK2) belongs to Ser/Thr protein kinase family which can promote S-phase initiation of cell cycle through the formation of functional complexes with Cyclin A and Cyclin E^27,28^. Upregulation of CDK2 expression can be found in various solid tumors and is closely related to the formation and development of tumors^29–31^. DNA damage increases the p53 protein levels and upregulates the p21 protein which can bind to and inhibit CDK2 kinase^32^. CDK2 can also phosphorylate p53 and activate the downstream signal transduction pathways^33^.

In the HCC patient tumor tissues, as well as GEO (GSE6764, GSE36376, GSE45436, and GSE62232) and TCGA datasets analyzed in this study, significant upregulation of SKA3 was detected, indicating poor prognosis. Knockdown of SKA3 affects cell invasion and proliferation both in vitro and in vivo. Gene set enrichment analysis (GSEA) results suggested that there is a correlation between SKA3 expression and the cell cycle and also the p53 signaling pathway. Flow cytometry showed that the depletion of SKA3 resulted in G2/M phase arrest and severe apoptosis. Mechanistically, knockdown of SKA3 promoted the phosphorylation of CDK2/P53 and upregulated BAX/Bcl-2 level. In this study, we demonstrated that in HCC, tumor progression is promoted by SKA3 through its action on the regulation of CDK2/p53 phosphorylation.

## Materials and methods

### Clinical patient specimens and information

All tissue samples used in this research were provided by HCC patients at the First Affiliated Hospital of Sun Yat-sen University who had undergone hepatectomy from July 2013 to December 2015. Radiotherapy or chemotherapy was not received by any of these patients before the operation and follow-up continued up to November 2018. Patient clinical characteristics are summarized in Table 1. Ethical and legal standards dictated that anonymous acquisition and processing methods are followed for all specimens. After resection, liquid nitrogen was used to rapidly freeze and store the fresh tumor tissue specimens at a temperature of −80 °C. The overall survival (OS) is taken to indicate the time from surgery to death or final contact for any reason (i.e., patients lost during follow-up were follow-ups of the patients who survived continued until the end of the study period). Disease-free survival (DFS) is taken to indicate the time from surgery until the first recurrence/metastasis of tumor or death due to any cause (i.e., patients lost during follow-up were follow-ups of the patients who survived continued until the end of the study period). Patients who survived without tumor recurrence and metastasis were followed up for the last time.

**Table 1:** Correlation between SKA3 expressions with clinic-pathological characteristics of HCC.

Clinicopathological Variables	*N*	SKA3 expression		*P-V*alue
		Low (102)	High (134)	
Sex				0.244
Male	120	55	65	
Female	116	47	69	
Age, years				0.438
<50	139	59	80	
≥50	97	43	54	
AFP, ng/L				**8.856e−11**
<200	153	89	64	
≥200	83	13	70	
HBsAg				0.398
Negative	82	34	48	
Positive	154	68	86	
Tumor size, cm				**3.29e–11**
≤5	138	84	54	
>5	98	18	80	
Tumor nodule number				**8.29e−07**
Solitary	146	81	65	
Multiple (≥2)	90	21	69	
Cancer embolus				**5.83e−14**
Absence	152	92	60	
Presence	84	10	74	
TNM stage				**1.6e−05**
Early (I & II)	157	83	74	
Late (III & IV)	79	19	60	
Differentiation grade				**0.002**
Well	173	85	88	
Poor	63	17	46	
P53 expression				**0.021**
High	95	33	62	
Low	141	69	72	

*AFP* alpha fetoprotein, *HBsAg* hepatitis B surface antigen

Bold values indicate statistical significance *p* < 0.05

### Data analysis of public databases

We acquired HCC expression data from GEO (datasets GSE62232, GSE45436, GSE36376, and GSE6764; http://www.ncbi.nlm.nih.gov/geo) and TCGA (http://gdc.cancer.gov/) databases. Log2 conversion was performed for all data in data standardization. EdgeR package was used to analyse gene differential expression for the data in count form. Limma package was used for the data in GEO database.

We selected genes whose |logFC (fold change)| > 1 and *p*-value < 0.05 as differential expression genes. Graphpad prism 7 was used to construct boxplots and scatter plots.

### Immunohistochemical staining and antibodies

There are 236 cases of formalin-fixed paraffin-embedded specimens used for SKA3 immunohistochemical staining. SKA3 antibodies used for immunohistochemistry staining were purchased from Bioss (bs-7848R). The samples were mixed with the primary anti-SKA3 polyclonal antibody and incubated overnight at 4 °C (dilution ratio 1:1000), after deparaffinization, hydration and blocking were performed. Thereafter, the staining of HCC was compared with that of normal specimen under microscope. They were divided into two groups: staining intensity score and positive cell score. The staining intensity scores were as follows: 0: no staining; 1: slightly yellow than the background; 2: yellow brown; 3: Brown. The positive cells were scored as follows: 0: 0–5%; 1: 6–25%; 2:26–50%; 3:51–75%; 4: >75%. The immunohistochemical score was calculated by multiplying the positive cell score by the staining intensity score. The final score was assigned four grades: negative (−) 0, weak positive (+) 1–4, positive (++) 5–8 and strong positive (++) 9–12.

### RNA extraction and quantitative real-time polymerase chain reaction (qRT-PCR)

Total RNA was isolated using TRIzol reagent (USA, NY, USA), following the manufacturer's instructions. QRT-PCR was performed using the following SKA3 primers on a SYBR Green detection RT-PCR system (TaKaRa, Japan):

Sensitive primers: CAGATCCCTCTTCACCTACGA;

Antisense primers: TCAACGTTTAAAGGGGGACA;

GAPDH (forward primers: TGTGTGTGGCATCAATGGATTTGG and reverse primers: ACACCATGTATTCCGTCAAT) (Servicebio Technology, Wuhan, China). The reference control used was GAPDH. The 2^−ΔΔCt^ method was used to quantitatively analyze relative mRNA expression levels. Three independent experiments of all qRT-PCR procedures were performed.

### Cell culture and transfection

Human hepatocellular carcinoma cell lines (LM3 and HUH7) were cultured in American-type media (ATCC, Manassas, VA) at a humid atmosphere of 5% CO_2_ with DMEM supplemented with 10% fetal bovine serum (penicillin and streptomycin) at 37 °C. In order to inhibit the expression of SKA3, we transfected three small interfering RNAs (siRNAs) targeting SKA3 coding sequence. The siRNAs sequences are as follows:

1. GGAAGAGCCCGUAAUUGUA
2. GAUCGUACUUCGUUGGUUU
3. AAUCCAGGCUCAAUGAUAA

Shanghai Generay Biotechnology Co., Ltd. had constructed the SKA3 knockdown vector, which was mixed with pPACKH1 packaging plasmid and transfected into 293TN cells with sh-SKA3/NC. 3 days later, according to the SBI packaging plan, the virus particles were collected from letinous edodes concentrated virus precipitation solution. TUNDUX virus transducers were used to infect cells. The target sequences are as follows:

1. GATCTGTCTGATCCTCCTGTT
2. CCTCTTCACCTACGATTTCTT

Puromycin screening was conducted to identify positive cells.

### Western blot and antibodies

PBS at 4 °C was used to wash the cells twice. Thereafter, protease inhibitors in cold RIPA buffer were used to lyse the cells. Protein concentration was determined using BCA Protein Quantitation Assay (KeyGen Biotech, Nanjing, China). Total protein of the cells were denatured using 10% SDS-PAGE and then transferred onto a nitrocellulose membrane. 5% non-fat milk in Tris-buffered saline containing 0.1% Tween-20 (TBST) was used to block the membrane for 1 h at room temperature. The membranes were then incubated with the primary antibodies overnight at 4 °C (dilution ratio 1:2000). TBST was used to wash the membranes three times and they were subsequently incubated with secondary antibodies (anti-rabbit IgG or anti-mouse IgG) for 1 h at room temperature. TBST was once again used to wash the membranes three times. The targeted proteins were identified using the ECL (EMD Millipore, MA, USA) method. SKA3, CCNE2, CCNA2, CDK4, CDK2, P53, P53-pSer15, P53-pSer46 antibodies used for western blot in this research were purchased from Bioss (bs-7848R), Abcam (ab32147), Abcam (ab108357), Abcam (ab181591), Abcam (ab32103), Abcam (ab32389), CST #9284 and CST#2521.

### Cell proliferation assay

HCC cell proliferation was analyzed using Cell count KIT-8 assay (CCK8, Dojindo, Tabaru, Japan). After transfection, the cells were added into 96-well plates (2000 cells/plate). Then, the cells were incubated and 450 nm absorption value was recorded at 24, 48, 72, and 96 h respectively. Measurements were performed in triplicate and the results are shown as an average of +SD.

### Cell invasion assay

Transwell chambers were to detect the invasion ability of HCC cells. 50 mg/L Matrigel 1:8 diluent was used to coat the upper chamber surface of the bottom membrane of the Transwell chamber, which was then air-dried at 4 °C. 200 μL of the cell suspension was taken and included into the mixture in the Transwell chamber (100,000 cells for each chamber) and cultured for 24 h. The data are shown as mean ± SD.

### Flow cytometry

HCC cells were isolated, fixed in 75% ethanol and stored overnight at 4 °C, for the cell cycle analysis. Thereafter, DNA Prep (Beckman Coulter, Brea, CA, USA) was used to stain the cells, and the percentage of cells at different stages was determined by flow cytometry, based on DNA content.

### Scratch wound healing assay

After seeding the HCC transfected cells into 6-well plates, the cells were incubated in 5% CO_2_ at 37 °C, until 100% confluency was reached. Thereafter, the monolayer was scraped with a 10 L pipette. After removing the cell debris, cell culture was continued under normal conditions. Photos were taken at 0 h and 24 h, show the relative distance between two edges.

### Plate clone formation assay

600 cells were added into each well of a 6-well plate and culture continued for 12 days to prepare the cells for the plate clone formation assay. The cultured medium was changed every 4 days. Then, the cells were fixed in 4% formaldehyde and crystal violet was used to stain the cells. Cell clones were counted and analyzed.

### Animal experiments

Animal experiments were approved by the Institutional Assessment Committee of Sun Yat-sen University and followed Institute Animal Care and Use Committee (IACUC) of Sun Yat-sen University guidelines. A subcutaneous xenograft model was established by subcutaneously injecting nude mice with 2 × 10^6^ LM3 cells on the right side. The tumor volume was measured with calipers were used to measure tumor volume and was repeatedly measured every 7 days (length × width2)/2. Twenty-eight days following implantation, in order to perform cervical dislocation, the mice were euthanized. Then, the xenografts were removed, fixed, weighed, photographed and preserved. Ki-67 antibodies for immunohistochemistry staining was obtained from Bioss (bs-2130R).

In order to establish in vivo lung metastasis model, 1 × 10^6^ cells were intravenously injected into the lateral tail vein of nude mice (*n* = 5 per group). The mice were measured using A Berthold bioluminescence system was used to measure the mice, and at week 8 the mice were sacrificed. Thereafter, in order to analyze the presence of metastatic nodules, the lungs were fixed, photographed, preserved and H&E stained.

All 4-week old male Balb/c nude mice used in this study were purchased from Nanjing Biomedical Research Institute, Nanjing University. Assignment of the mice into different groups was done randomly. Investigators were not blinded to group allocation.

### GSEA

In order to identify gene sets and pathways correlated with SKA3 data obtained from TCGA was used to perform a GSEA, in which the expression of each gene on the list is weighted based its log fold change. GSEA software was downloaded for use from the Broad Institute Website (http://software.broadinstitute.org/gsea/index.jsp).

### Co-Immunoprecipitation

Overexpressed Flag- or HA-labeled cell protein extracts were incubated with antibodies (or IgG as control, Sigma) and binding protein A/G beads (Pierce) for 12 h at 4 °C. After washing three times with an IP buffer, western blotting was used to analyze the samples.

### Statistical analysis

All data analysis in this article was conducted using R software. For categorical variables of HCC patients, we used Pearson correlation coefficients analysis. Independent risk factors among HCC patients were identified using the univariate and multivariate Cox proportional hazards model. The effect of SKA3 on the prognosis of the HCC patients was evaluated using the Kaplan–Meier method, and the survival curves were established by survival package. For continuous data obtained from in vitro and in vivo experiments, significant differences were determined using the Student’s *t*-test. All experiments in this research were independently repeated at least three times. We consider statistical difference with *P* < 0.05.

## Results

### SKA3 is upregulated in human hepatocellular carcinoma and indicates poor prognosis

**Fig. 1:**
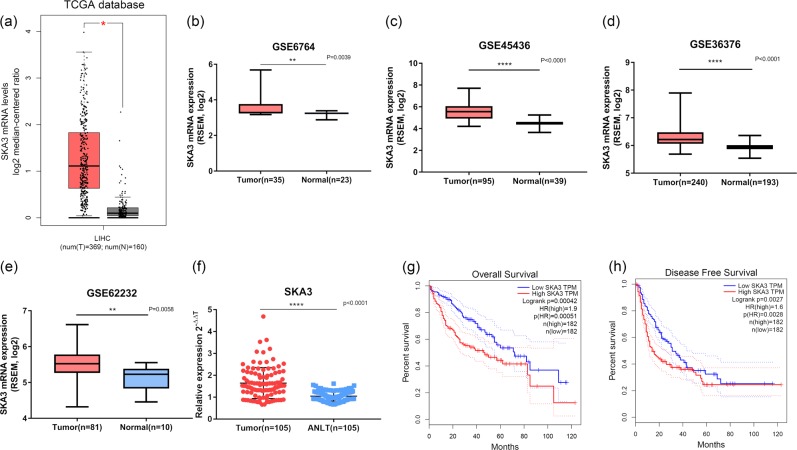
SKA3 is upregulated in HCC tissues compared with ANLTs. SKA3 was highly expressed in HCC tissues compared with normal liver tissues according to analysis of data from TCGA and GETx database **a** tumor, *n* = 369; normal = 160, *P < 0.01) and GEO **b**–**e** GSE6764, tumor, *n* = 35; normal, *n* = 23, **P = 0.0039; GSE45436, tumor, *n* = 95; normal, *n* = 39, ****P < 0.0001; GSE36376, tumor, *n* = 8240; normal, *n* = 193, ****P < 0.0001; GSE62232, tumor, *n* = 81; normal, *n* = 10, **P = 0.0058). **f** Real-time PCR analysis of SKA3 expression in 105 pairs of HCC specimens and corresponding ANLTs (***P = 0.0005). **g**–**h** Overall survival and disease-free survival analysis of TCGA. (All *P < 0.05, **P < 0.01, ***P < 0.001, ****P < 0.0001.

We acquired HCC patients’ gene expression and corresponding clinical data from TCGA database and GEO (GSE62232, GSE45436, GSE6764, and GSE36376). SKA3 was identified to be elevated in all data sets, Fig. 1 shows the detailed results of this analysis. Real-time PCR was performed on 105 pairs of fresh tumor tissues and adjacent normal liver tissues (ANLT), and the result confirmed the upregulation of SKA3 in HCC tissues (Fig. 1f). Then we performed Kaplan–Meier analysis using TCGA datasets and the results showed that patients with overexpression of SKA3 have shorter OS and DFS (Fig. 1g, h). This suggests that the expression of SKA3 is elevated in human hepatocellular carcinoma and positively correlated with the malignancy of the tumor.

### Altered SKA3 expression affects cell proliferation in vivo and in vitro

**Fig. 2:**
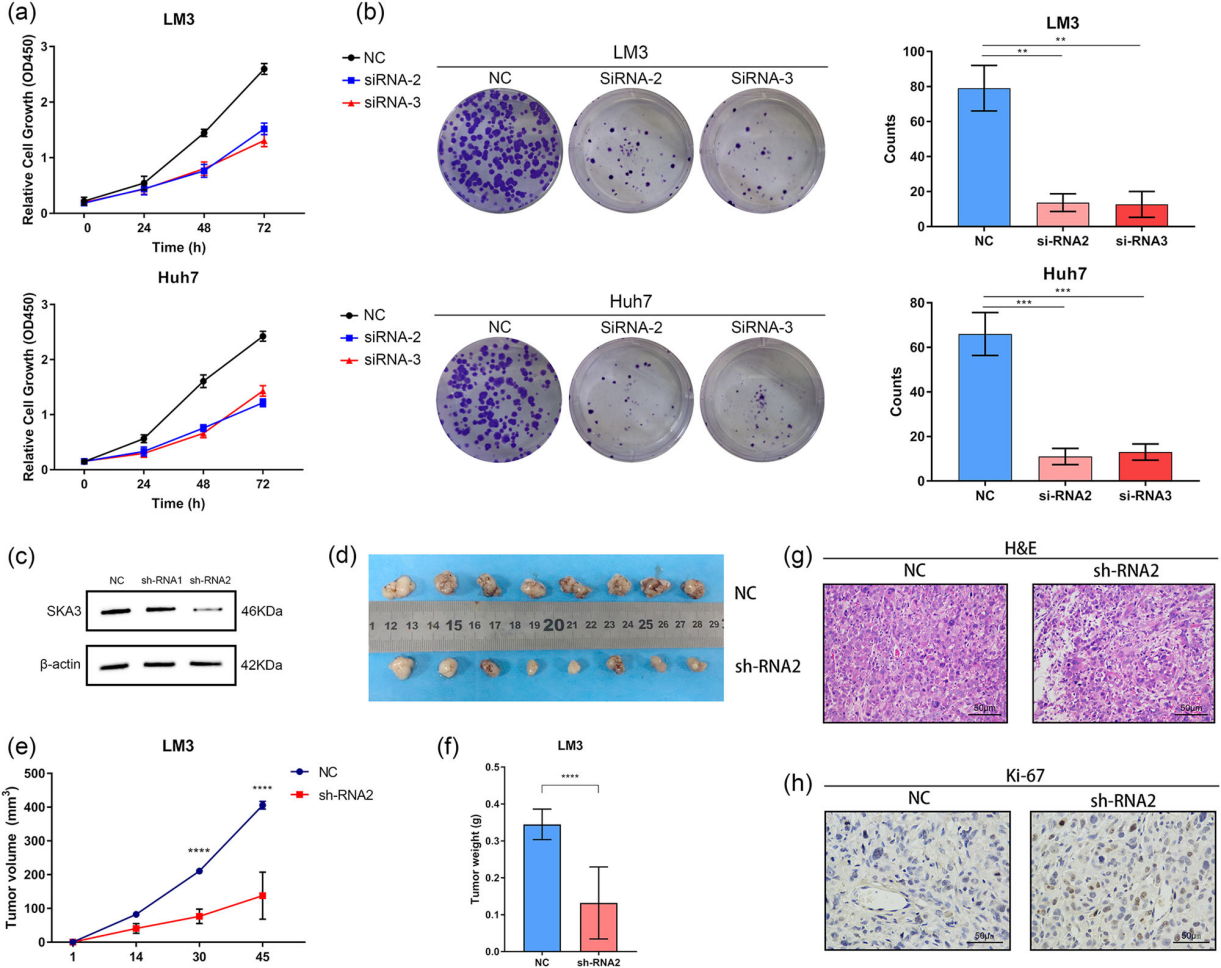
Inhibition of SKA3 affects cell proliferation in vitro and in vivo. **a** CCK8 and **b** colony-forming assays were used to determine the cell viability of LM3 and Huh7 cells co-transferred with SKA3 siRNAs. The number of colonies per well was counted. **c** Western blot analysis of SKA3 expression after SKA3 silencing in LM3 cells. **d** Nude mice were sacrificed forty-five days after the injection and tumors from respective groups were shown (*n* = 10/group). **e** Tumor growth curves after the injection of LM3 cells. Tumor volume was calculated every 7 days. **f** Tumor weight was measured in sh-RNA2 and control groups (****p < 0.0001). **h** H&E staining of tumors with different treatments. Scale bar: 50 μm. **g** IHC staining of Ki67 in tumors with different treatments. Scale bar: 50 μm. All *P < 0.05, **P < 0.01, ***P < 0.001, ****P < 0.0001.

In order to study the role of SKA3 in hepatocellular carcinoma (HCC), three kinds of siRNA targeting SKA3 (siRNA-1, siRNA-2, and siRNA-3) were transfected into LM3 and Huh7 cell lines, and siRNA was transfected as control. The expression of SKA3 in the cells transfected with siRNA2 and siRNA3 were significantly lower than that in the control group (Fig. S1). Therefore, we used siRNA2 and siRNA3 transfected cells for experiments. CCK8 assay and plate clone formation assay show that in LM3 and Huh7 cell lines, SKA3 downregulation inhibits cell proliferation (*P* < 0.05; Fig. 2a, b). A SKA3-stable knockdown LM3 cell line was constructed using lentiviruses carrying shRNA (Fig. 2c) to detect whether SKA3 expression levels have an impact on tumor progression in vivo. SKA3 knockdown cells and control cells were subcutaneously injected into BALB/c nude mice. Tumor growth in SKA3 deficiency was slower than that in the control group (Fig. 2d, e). Euthanasia was performed in mice and the tumor was measured 45 days after injection. At the end of the experiment, the tumor weight of LM3 transfection group was significantly reduced (****P < 0.0001; Fig. 2f). In addition, significant downregulation of the proliferation marker gene Ki-67 was found in SKA3 knockdown tumors (Fig. 2h) using IHC staining. These results indicate that knockdown of SKA3 inhibits the development of tumor in vitro and in vivo.

### Elevated SKA3 levels promote tumor invasion in vivo and in vitro

**Fig. 3:**
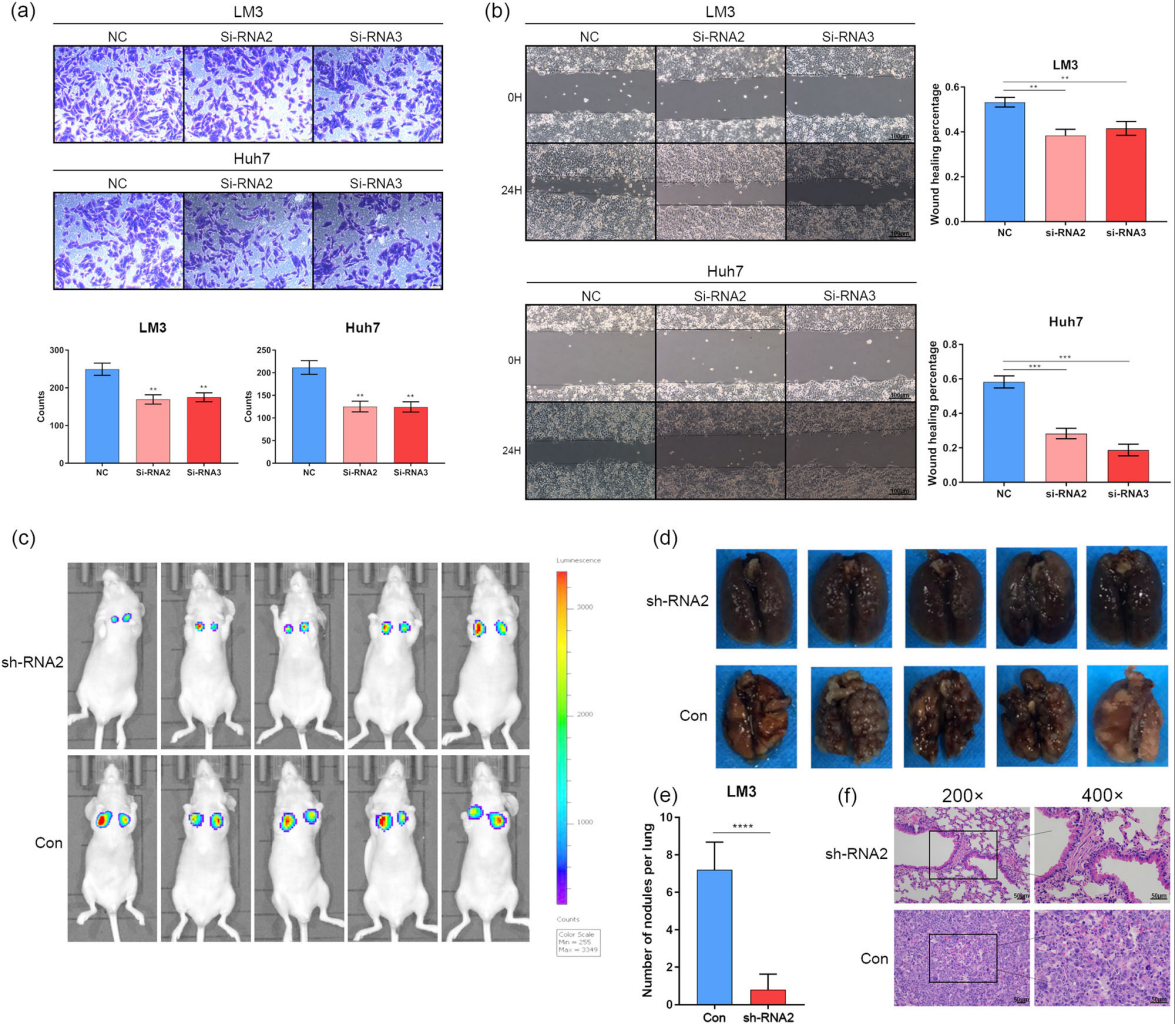
Depletion of SKA3 inhibits tumor invasion in vitro and in vivo. **a**, **b** Transwell and Scratch wound healing assays was performed to determine the cell motility of LM3 and Huh7 cells co-transferred with SKA3 siRNAs. Scale bar: 200 μm. **c** Deletion of SKA3 inhibits LM3 cells metastasis in vivo (*n* = 5/group). The bioluminescent change in LM3^shRNA−SKA3^ group was significantly decreased compared with the vector control. The quantitation of lung metastasis was assessed by bioluminescence measurements. **d** Representative photographs of gross lungs from indicated groups are shown (*n* = 5/group). **e** Number of lung metastatic nodules in indicated groups. **f** Representative H&E images of lung metastatic tumors from indicated groups. (*n* = 5/group). Scale bar: 100 μm. All *P < 0.05, **P < 0.01, ***P < 0.001, ****P < 0.0001.

Analysis of cell invasion in vitro was conducted using the Scratch wound healing test and transwell assay. In the transwell assay, the downregulation of SKA3 significantly decreased the invasive ability of Huh7 cells and LM3 cells (*P* < 0.05; Fig. 3a). In SKA3 knockdown groups, tumor cells were slower than the control cells in closing the wound at 24 h (*P* < 0.05; Fig. 3b). Then we constructed lung metastasis mouse model to study the function of SKA3 in vivo. The metastases in the LM3-shSKA3 group were obviously inhibited relative to the control 8 weeks after injection (Fig. 3c–e). Hematoxylin and eosin (H&E) staining of the lung sections of these mice verified the observations (Fig. 3f). These results indicate that SKA3 promotes tumor invasion both in vitro and in vivo.

### The regulatory function of SKA3 in on proliferation, cell cycle and apoptosis relies on P53

**Fig. 4:**
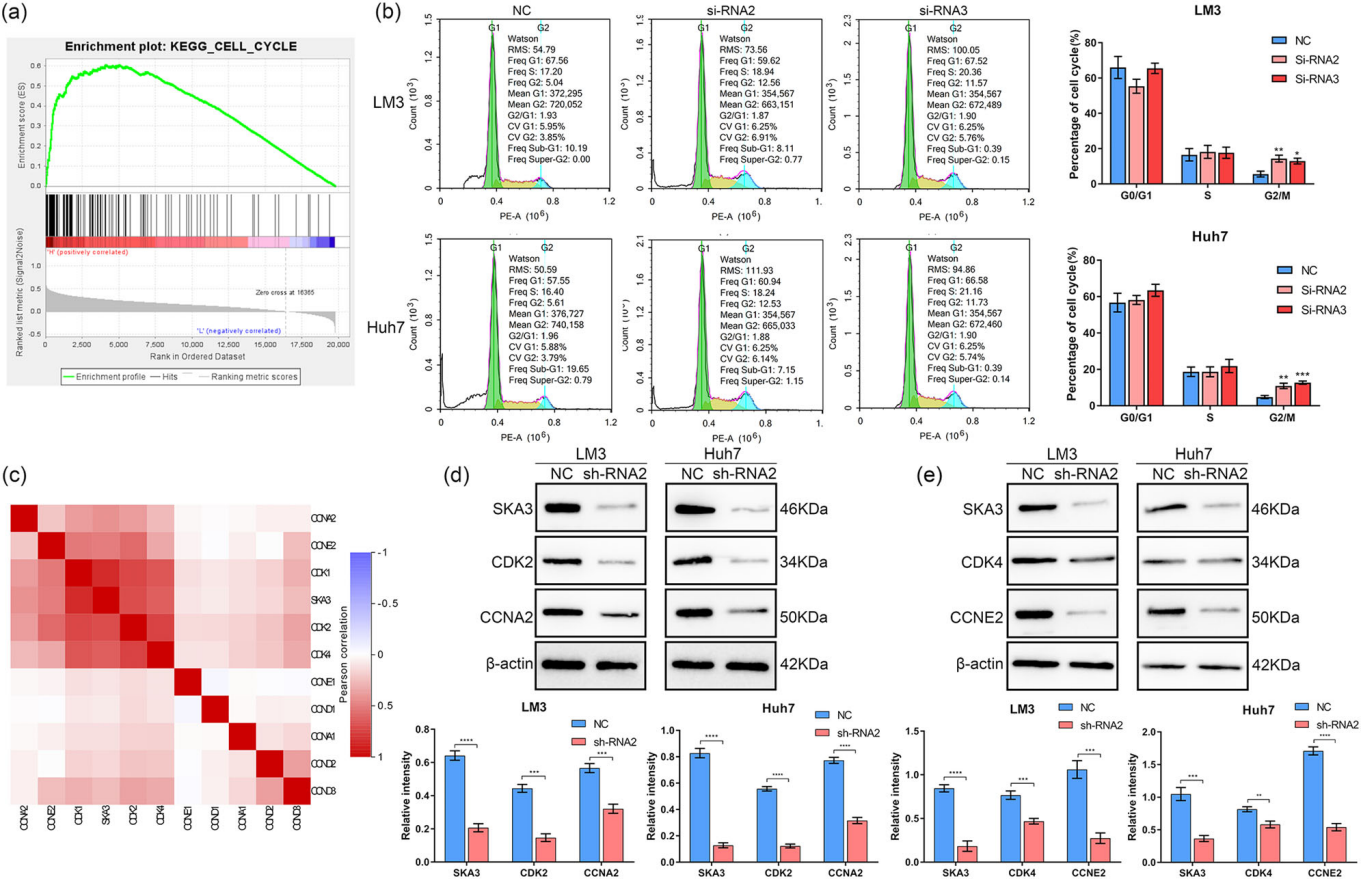
Downregulation of SKA3 affects the cell cycle in HCC cells. **a** GSEA analysis suggested that SKA3 is related to cell cycle in HCC using TCGA datasets. **b** Depletion of SKA3 triggered G2 block in LM3 and Huh7 cells. **c** Correlation analysis showed that SKA3 is strongly related with CDK1, CDK2, CDK4, CCNA2, and CCNE2. **d**, **e** Western blot analysis suggested that knockdown of SKA3 upregulated CDK2, CDK4, CCNA2, and CCNE2. All *P < 0.05, **P < 0.01, ***P < 0.001, ****P < 0.0001.

**Fig. 5:**
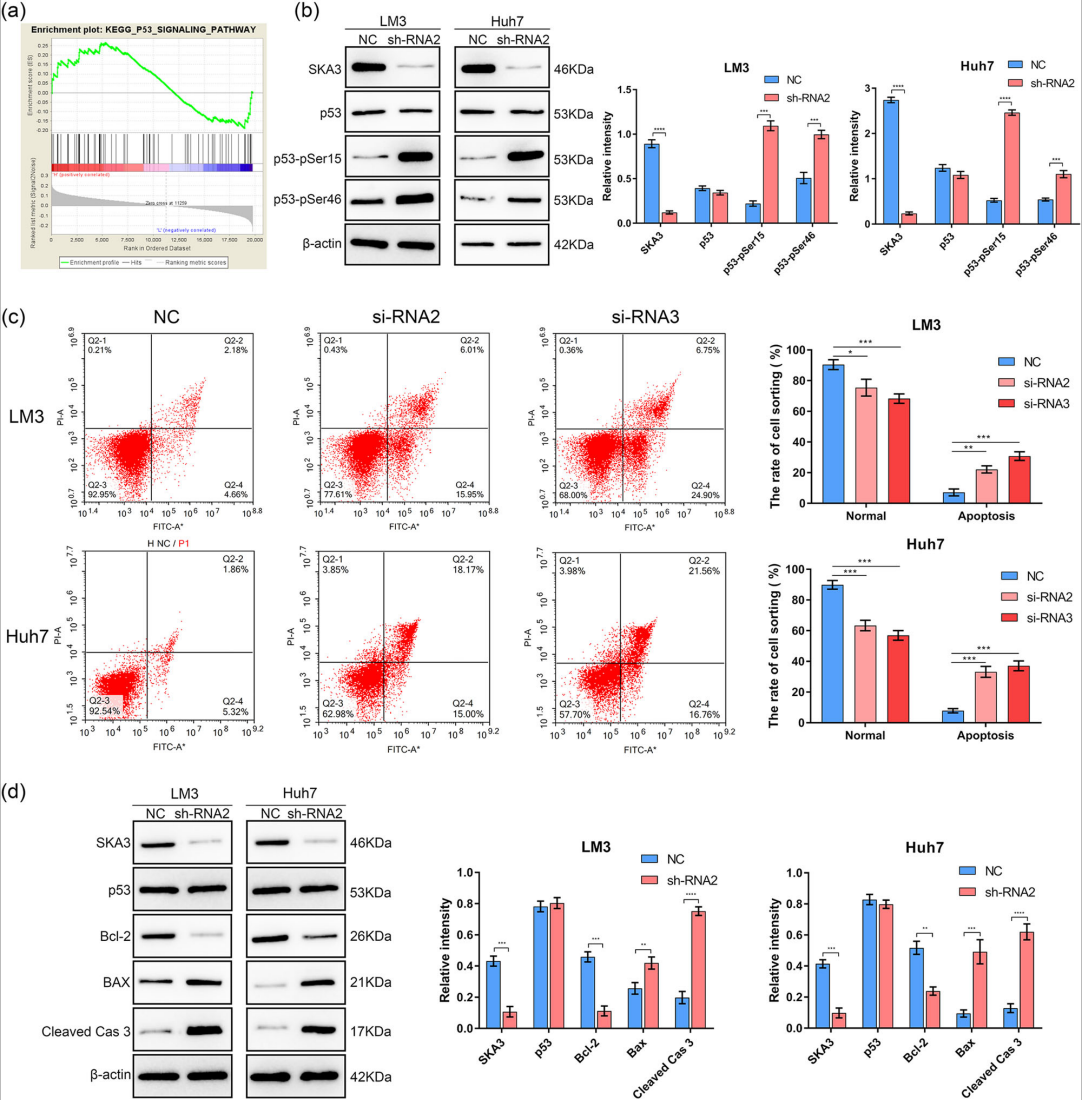
Depletion of SKA3 promotes apoptosis in HCC cells. **a** GSEA analysis suggested that SKA3 is related to P53 signaling pathway in HCC. **b** Western blot results showed that knockdown of SKA3 increased phosphorylation level of P53 in LM3 and Huh7 cells. **c** Flow cytometry was performed to determine the cell apoptosis in si-SKA3 groups. **d** Western blot analysis showed that knockdown of SKA3 upregulated BAX/Bcl-2 level in HCC cells. All *P < 0.05, **P < 0.01, ***P < 0.001, ****P < 0.0001.

The aim of this study was to explore the regulatory mechanism of SKA3 on the invasion and proliferation of hepatoma cells. As shown in Figs. 4a and 5a, SKA3 is significantly associated with cell cycle and P53 signaling pathway. SKA3 is most likely to influence tumor progression through regulating P53 related cell cycle progression and apoptosis. Western blot analysis showed that depletion of SKA3 promoted the phosphorylation of P53 at P53-pSer15 and P53-pSer46 (Fig. 5b). We supplemented cell proliferation, clone formation, Transwell, scratch, and flow cytometry experiments in P53-deficient hepatocellular carcinoma cell line Hep3B shown in Figure S2. No significant difference was found between the experimental group and the control group, because p53 could not be phosphorylated in the P53-deficient liver cancer cell lines. Therefore, SKA3 could regulate tumor growth in HCC through p53 signaling pathway.

### SKA3 participates in cell cycle regulation in HCC cells

Flow cytometry was used to study the effect of SKA3 on the cell cycle. As shown in Fig. 4b, the cell cycle of SKA3 siRNA group was arrested at G2 stage (*P* < 0.05). Then we used TCGA data to analyze the relationship between SKA3 and Cyclins and CDK. Pearson correlation coefficient was calculated and we found that the expressions of CDK4, CDK2, CDK1, CCNA2, and CCNE2 were positively correlated with SKA3 (*r*^2^ > 0.36) (Fig. 4c). Then we performed western blot and the results confirmed that the levels of CDK2, CDK4, CCNA2 and CCNE2 were lower in Sh2-SKA3 treated cells (Fig. 4d, e). SKA3 is strongly associated with cell cycle regulation in HCC.

### Knockdown of SKA3 promoted apoptosis by regulating BAX pathway

GSEA results suggested that SKA3 is related with P53 signaling pathway (Fig. 5a). Therefore, we then studied the role of SKA3 in p53 mediated apoptosis. Flow cytometry results showed that the depletion of SKA3 increased the percentage of apoptosis cells in LM3 and Huh7 cells (Fig. 5c). As is shown in Fig. 5d, the expression of cleaved caspase 3 were elevated in sh-SKA3 groups. Mechanically, depletion of SKA3 significantly upregulated BAX in HCC and inhibited Bcl-2 in HCC cells (Fig. 5d). SKA3 could promote the apoptosis resistance of HCC by decreasing BAX/Bcl-2 level.

### SKA3 inhibited the interaction between CDK2 and p53

**Fig. 6:**
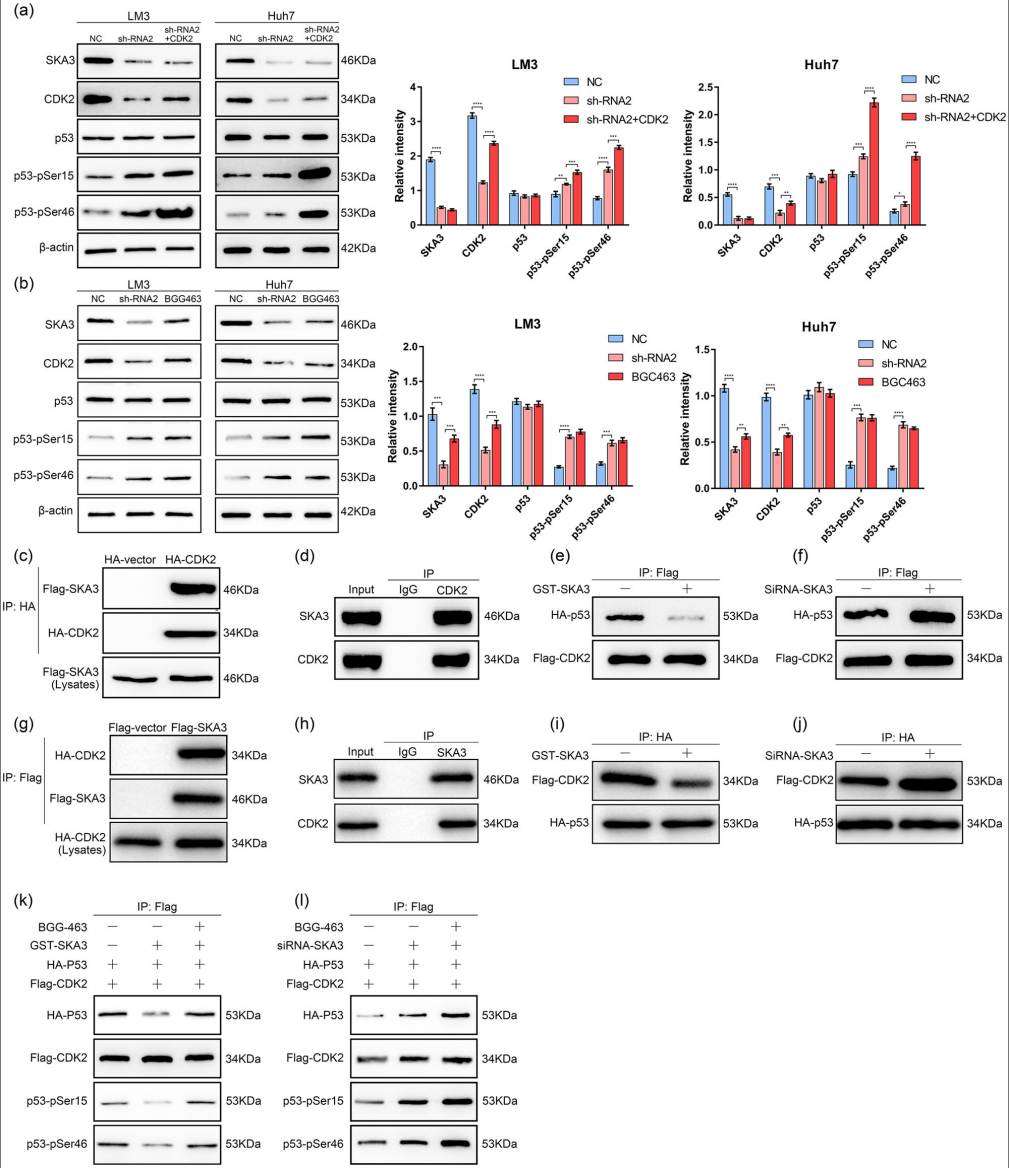
SKA3 inhibited the interaction between CDK2 and p53. **a** Western blot analysis of SKA3, CDK2, p53, and phosphorylated p53 in sh-SKA3, sh-SKA3+ CDK2 and control groups. The ratio shows relative protein expression normalized for β-actin. **b** Western blot analysis of SKA3, CDK2, p53, and phosphorylated p53 in sh-SKA3, BGG463 and control groups. **c**, **g** Total lysates from HEK193T cells expressing Flag-SKA3 and HA-CDK2 were subjected to IP with HA Ab or Flag Ab, followed by western blotting using the indicated antibodies (Abs). HA-P53 and Flag-CDK2 were used as a loading control. **d**, **h** Total lysates from HEK293T cells expressing SKA3 or CDK2 were subjected to IP with SKA3 Ab or CDK2 Ab, followed by western blotting using the indicated Abs. **e**, **i** Total lysates from HEK293T cells expressing HA-P53 and Flag-CDK2 in the presence of GST-SKA3 were subjected to IP with Flag Ab or HA Ab, followed by western blotting using the indicated Abs. **f**, **j** Total lysates from HEK293T cells expressing HA-P53 and Flag-CDK2 in the presence of siRNA-SKA3 were subjected to IP with Flag Ab or HA Ab, followed by western blotting using the indicated Abs. **k** Total lysates from HEK293T cells expressing HA-P53 and Flag-CDK2 in the presence of GST-SKA3 and BGG463 were subjected to IP with Flag Ab, followed by western blotting using the indicated Abs. **l** Total lysates from HEK293T cells expressing HA-P53 and Flag-CDK2 in the presence of siRNA-SKA3 and BGG463 were subjected to IP with Flag Ab, followed by western blotting using the indicated Abs. All *P < 0.05, **P < 0.01, ***P < 0.001, ****P < 0.0001.

Then we studied the specific mechanism of SKA3 regulating p53 phosphorylation. For 48 h, the LM3 and Huh7 cells were transfected with an indicated quantity of siRNA and plasmids. Western blotting with indicated antibodies were used to analyze the cell lysates. The ratio shows relative SKA3, CDK2, p53, and phosphorylated p53 protein expression normalized for β-actin. Western blot results showed that SKA3 affects P53 phosphorylation at P53-pSer15 and P53-pSer46 (Fig. 5b). To further investigate the correlations between SKA3, CDK2, and P53, we constructed sh-SKA3+ CDK2 cells. Phosphorylation level of P53 was increased in sh-SKA3+ CDK2 group (Fig. 6a) while that was no difference in CDK2 inhibitor BGG463 group (Fig. 6b). Co-Immunoprecipitation (Co-IP) results showed that exogenous or endogenous SKA3 co-precipitated with exogenous or endogenous CDK2 in HEK293T cells. (Fig. 6c, d, g, h). We further tested whether SKA3 affects the stability of the CDK2/P53 complex. CO-IP experiments showed that the binding capacity of CDK2 or P53 was decreased when SKA3 was upregulated in HEK293T cells (Fig. 6e, i); meanwhile, the binding capacity of CDK2 or P53 was increased when SKA3 was suppressed in HEK293T cells (Fig. 6f, j). Then we used BGG463 to suppress the activation of CDK2. CO-IP results showed that the binding capacity of CDK2 was reversed in presence of BGG463 after overexpression of SKA3 (Fig. 6k), whereas the inactivation of CDK2 did not affect the binding capacity of CDK2 when SKA3 was suppressed in HEK293T cells (Fig. 6l). We supplemented the CO-IP experiments in LM3 cells and the results were shown in Fig. S3. These results indicate that SKA3 inhibited the activation of p53 by interfering the interaction between CDK2 and p53.

### Overexpression of SKA3 in HCC patients predicts poor survival

**Fig. 7:**
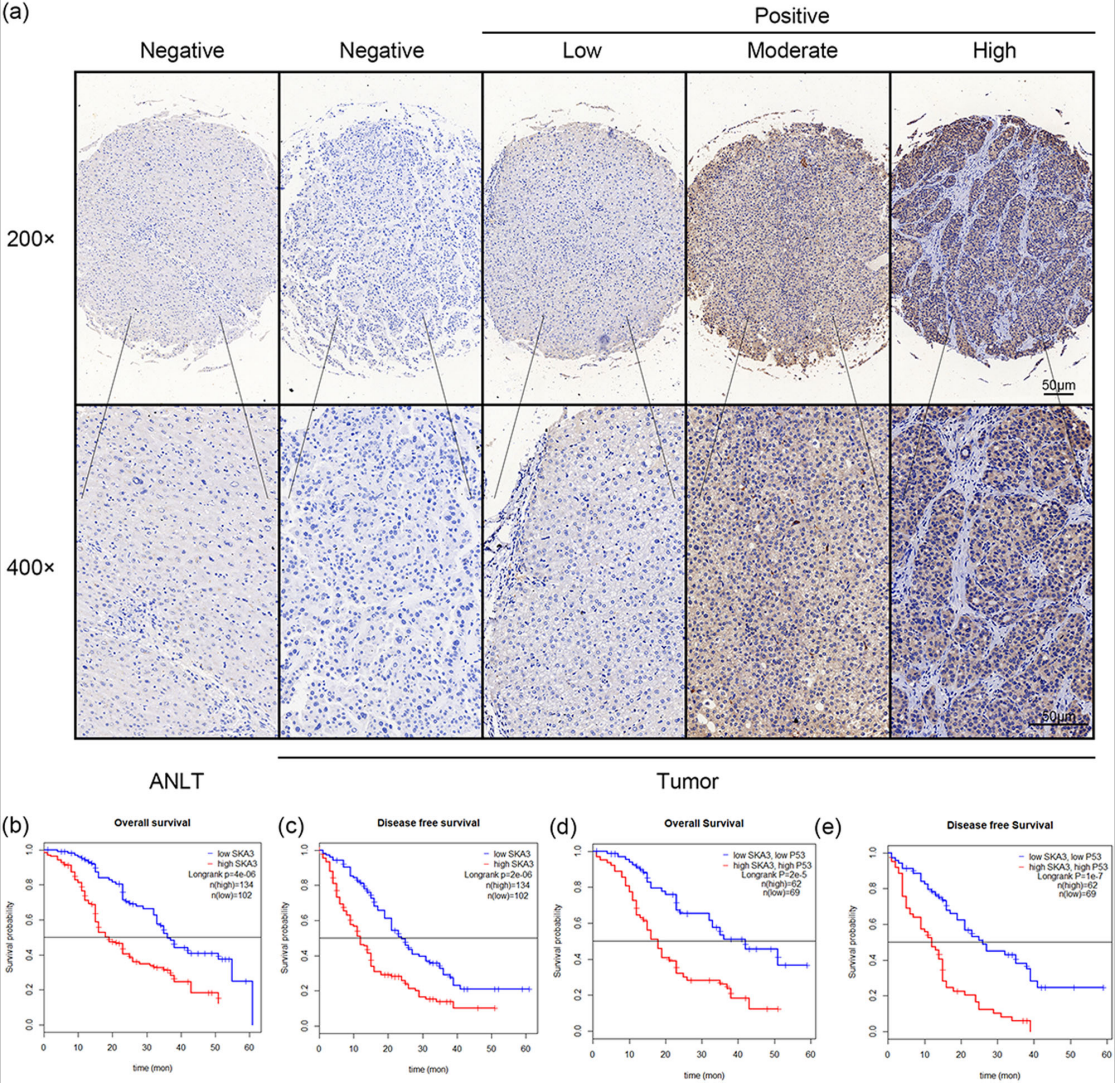
SKA3 expression is correlated with clinicopathological features and poor prognosis. **a** Immunohistochemical staining showed elevated SKA3 expression in HCC tissues compared to corresponding normal liver tissues in 236 pairs of clinical samples. Scale bars: 100 μm. **b**, **c** Overall survival and disease-free survival curves showed that patients with high expression levels of SKA3 have worse prognosis. **d**, **e** Overall survival and disease-free survival curves showed that patients with high expression levels of SKA3 and P53 have worse prognosis.

Immunohistochemical results showed that 134 out of 236 HCC patients had higher levels of SKA3 expression and 102 had lower levels of SKA3 expression (Fig. 1i). The correlation between SKA3 expression and clinical features of hepatocellular carcinoma is shown in Table 1. SKA3 expression was positively correlated with AFP (*P* < 0.0001), tumor size (*P* < 0.0001), tumor nodule number (*P* < 0.0001), tumor thrombus (*P* < 0.0001), TNM stage (*P* < 0.0001), differentiation degree (*P* = 0.002) and P53 expression (*P* = 0.021). The DFS and OS of patients with high level of SKA3 expression were worse in our clinical patients (Fig. 1j, k). We then used immunohistochemical staining to explore the status of P53 in those patients. The results showed that 95 out of 236 patients had higher levels of SKA3 expression and 141 had lower levels of SKA3 expression. Kaplan–Meier analysis found that patients with high expression of SKA3 and P53 have shorter OS and DFS (Fig. 7d, e). It was explored whether SKA3 is an independent prognostic factor in HCC patients using a Cox proportional hazard model. Multivariate analysis results confirmed that SKA3 expression is an independent risk factor for DFS (Tables 2 and 3). Thus, SKA3 can served as an independent biomarker for prognosis of patients with liver cancer.

**Table 2:** Univariate and multivariate Cox regression analysis of risk factors associated with overall survival.

Variables	Univariate analysis			Multivariate analysis		
	HR	95% CI	*P-*Value	HR	95% CI	*P-*Value
SKA3 expression (high vs. low)	2.239	1.574–3.187	**7.59e−06**	1.299	0.829–2.034	0.25
Sex (male vs. female)	0.935	0.67–1.305	0.69			
Age (≥50 vs. <50)	0.899	0.659–1.226	0.50			
HBsAg (positive vs. negative)	0.944	0.671–1.329	0.74			
AFP (≥200 ng/ml vs. <200 ng/ml)	1.797	1.279–2.525	**7.31e−04**	1.048	0.692–1.586	0.83
Tumor size (>5 cm vs. ≤5 cm)	2.441	1.742–3.421	**2.19e−07**	1.688	1.136–2.509	**0.0095**
Tumor nodule number (multiple vs. single)	1.797	1.284–2.514	**6.27e−04**	1.086	0.744–1.585	0.67
Cancer embolus (presence vs. absence)	2.875	2.035–4.061	**2.07e−09**	1.878	1.231–2.866	**0.0035**
TNM stage (late vs. early)	2.095	1.485–2.956	**2.5e−05**	1.209	0.804–1.818	0.36
Differentiation grade (poor vs. well)	1.545	1.08–2.211	**0.017**	0.970	0.648–1.452	0.88

*AFP* alpha fetoprotein, *HBsAg* hepatitis B surface antigen

Bold values indicate statistical significance *p* < 0.05

**Table 3:** Univariate and multivariate Cox regression analysis of risk factors associated with disease-free survival.

Variables	Univariate analysis			Multivariate analysis		
	HR	95% CI	*P-*Value	HR	95% CI	*P-*Value
SKA3expression (high vs. low)	2.107	1.54–2.883	**3.18e−06**	1.501	1.017–2.216	**0.04**
Sex (male vs. female)	1.107	0.818–1.498	0.51			
Age (≥50 vs. <50)	0.912	0.67–1.24	0.56			
HBsAg (positive vs. negative)	1.441	1.04–1.998	**0.028**	1.745	1.234–2.466	**0.0016**
AFP (≥200 ng/ml vs. <200 ng/ml)	1.623	1.184–2.225	**2.63e−03**	1.042	0.707–1.536	0.83
Tumor size (>5 cm vs. ≤5 cm)	2.098	1.549–2.84	**1.66e−06**	1.667	1.174–2.365	**0.0042**
Tumor nodule number (multiple vs. single)	1.517	1.114–2.066	**8.13e−03**	1.077	0.76–1.525	0.68
Cancer embolus (presence vs. absence)	2.420	1.766–3.316	**3.88e−08**	2.002	1.046–3.832	**0.0362**
TNM stage (late vs. early)	1.451	1.049–2.008	**0.025**	1.051	0.715–1.547	0.80
Differentiation grade (poor vs. well)	1.480	1.064–2.058	**0.02**	0.909	0.62–1.333	0.62

*AFP* alpha fetoprotein, *HBsAg* hepatitis B surface antigen

Bold values indicate statistical significance *p* < 0.05

## Discussion

SKA3 is an important subunit of the spindle and centromere-associated protein complex^11^, located on chromosome 13q12.11, which controls and regulates mitosis with NDC80 complex, and also regulates cell proliferation and apoptosis^12^. In this study, we found that the expression level of SKA3 in liver cancer tissues and cells was significantly higher than that in normal liver tissues and cells, suggesting that SKA3 was highly expressed in liver cancer. Our study successfully downregulated SKA3 in LM3 and Huh7 cell lines. Transwell assays and orthotopic xenografts showed that knockdown of SKA3 reduces HCC cells invasion. Through cell clonal formation and CCK8 assay, we found that the proliferation of hepatoma cells after SKA3 overexpression was significantly enhanced, while the proliferation of hepatocarcinoma cells was significantly attenuated after SKA3 interference expression, suggesting that SKA3 can promote the proliferation of hepatoma cells.

Hinchcliffe et al. found that cytokine-dependent kinase 2 (CDK2) was required for cell centrosome replication, and that CDK2 allowed cells to pass through the cell division cycle, helping to ensure that the centrosome replicates only once in a positive time^34^. CDK2 binds to Cyclin E or Cyclin A and has been active in G1/S and M phases, respectively^35,36^. The expression level of CDK2 remains unchanged throughout the cell cycle. CDK2 is a key kinase that initiates DNA replication and is an essential factor in G2 phase of action. The results suggest that CDK2 and Cyclin E are involved in the cell entry from the G1 phase to the S phase. The CDK2/Cyclin E kinase complex plays a decisive role in driving the cell cycle from G1 to S phase. This complex is also required for centrosome replication. The activity of CDK2 is directly related to the cleavage of centrosomes^37^. In this study, bioinformatic analysis found that SKA3 has an important relationship with CDK2, and after knocking down SKA3, CDK2 was the major downregulated protein, which explained the G2 arrest of liver cancer cells after knocking down SKA.

The most basic biological feature of malignant tumors is the uncontrolled proliferation of tumor cells, and the biological basis of uncontrolled proliferation of cells is the disorder of cell cycle regulation^38^. Cell cycle regulation is a delicate biological process involving the involvement of multiple genes and proteins, of which p53 plays an important role in monitoring cellular genome damage and maintaining genome stability^39^. On the one hand, p53 protein is regulated by transcriptional regulation of genes such as p21, 14-3-3σ, GADD45, and Cyclin B1, and is monitored by cell G1 and/or G2/M phase checkpoints to induce cell cycle arrest and repair cell damage DNA^40^; on the other hand, p53 activates apoptosis-promoting genes such as Bax, Noxa, Puma, and Perp by transcription, induces apoptosis and prevents malignant transformation^41^. P53 not only causes cell cycle arrest, but also participates in apoptotic responses, resulting in two distinct results, the former providing the cell with the possibility of initiating repair and reversing the damage. Therefore, p53 is considered to be one of the key factors determining cell survival. Once activated, the transcriptional activity and target gene expression of p53 is enhanced following its phosphorylation by Cdk5 at Ser-46, Ser-33, and Ser-15^42^. Here, we first found that CDK2 phosphorylated the Ser-15 and Ser-46 of p53. At the same time, it was also found that SKA3 could affect the phosphorylation level of p53. However, after knocking down CDK2, the ability of SKA3 to affect the phosphorylation level of p53 was greatly reduced. Therefore, we believe that SKA3 prevents the phosphorylation of p53 by CDK2. In addition, we found that SKA3 interacted with CDK2 by immunoprecipitation experiments, and overexpression of SKA3 inhibited the binding of CDK2 and p53, that is, SKA3 and CDK2, which were highly expressed in hepatoma cells, bound to each other and prevent the activation of p53 pathway, thereby promoting liver cancer growth and invasion.

**Fig. 8:**
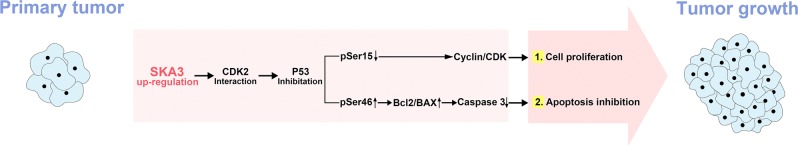
Schematic of the above-mentioned findings. SKA3 influences cell proliferation and apoptosis in LM3 and Huh7 cells via P53 signaling pathway. Furthermore, SKA3 affects the phosphorylation of P53 at P53-pSer15, and P53-pSer46 through inhibiting the interaction between CDK2 and P53.

In summary, SKA3 is highly expressed in liver cancer and can alleviate the apoptosis of liver cancer cells, while promoting proliferation, which may be related to inhabiting the phosphorylation of p53 by interaction with CDK2 (Fig. 8). The molecular mechanism of SKA3 promoting the proliferation of hepatoma cells will be further studied in the future, which will provide a solid foundation for the prevention and treatment of liver cancer.

## Supplementary information

table s1

supplementary figure legends

Figure s1

Figure s2

Figure s3

## Data Availability

The datasets used and/or analyzed during the current study can be obtained from the corresponding author on reasonable request.
