# Supplementary material for: SKA3 Promotes tumor growth by regulating CDK2/P53 phosphorylation in hepatocellular carcinoma
Source: Cell Death Dis. 2019 Dec 5;10(12):929. doi: 10.1038/s41419-019-2163-3 (PMC6895034; doi:10.1038/s41419-019-2163-3)
Supplement: Supplementary file 1 — table s1 [file 41419_2019_2163_MOESM1_ESM.docx]

**Table 1. Correlation between P53 expressions with clinic-pathological characteristics of HCC**

| Clinicopathological Variables | N | P53 Expression | | *P V*alue |
| --- | --- | --- | --- | --- |
|  |  | Low (141) | High (95) |  |
| **Sex** |  |  |  | 0.52 |
| Male | 120 | 72 | 48 |  |
| Female | 116 | 69 | 47 |  |
| **Age, years** |  |  |  | 0.438 |
| <50 | 139 | 84 | 55 |  |
| ≥50 | 97 | 57 | 40 |  |
| **AFP, ng/L** |  |  |  | 0.28 |
| <200 | 153 | 94 | 59 |  |
| ≥200 | 83 | 47 | 36 |  |
| **HBsAg** |  |  |  | 0.398 |
| Negative | 82 | 47 | 35 |  |
| Positive | 154 | 94 | 60 |  |
| **Tumor size, cm** | |  |  | **2.19e-04** |
| ≤5 | 138 | 96 | 42 |  |
| >5 | 98 | 45 | 53 |  |
| **Tumor nodule number** | |  |  | 0.122 |
| Solitary | 146 | 92 | 54 |  |
| Multiple (≥2) | 90 | 49 | 41 |  |
| **Cancer embolus** | |  |  | 0.153 |
| Absence | 152 | 95 | 57 |  |
| Presence | 84 | 46 | 38 |  |
| **TNM stage** |  |  |  | 0.224 |
| Early (I & II) | 157 | 97 | 60 |  |
| Late (III & IV) | 79 | 44 | 35 |  |
| **Differentiation grade** | |  |  | **0.033** |
| Well | 173 | 110 | 63 |  |
| Poor | 63 | 31 | 32 |  |
| **SKA3 expression** | |  |  | **0.021** |
| High | 102 | 69 | 33 |  |
| Low | 134 | 72 | 62 |  |

**Abbreviation: AFP, alpha fetoprotein; HBsAg, hepatitis B surface antigen.**
