# Supplementary material for: SKA3 Promotes tumor growth by regulating CDK2/P53 phosphorylation in hepatocellular carcinoma
Source: Cell Death Dis. 2019 Dec 5;10(12):929. doi: 10.1038/s41419-019-2163-3 (PMC6895034; doi:10.1038/s41419-019-2163-3)
Supplement: Supplementary file 2 — supplementary figure legends [file 41419_2019_2163_MOESM2_ESM.docx]

**Supplementary Figure Legends**

**Figure S1** Three kinds of siRNA targeting SKA3 (siRNA-1, siRNA-2 and siRNA-3) were transfected into LM3 and Huh7 cell lines, and siRNA was transfected as control. (a) The expression of SKA3 were lower in si-RNA2 and si-RNA3 groups in LM3 cells. (b) The expression of SKA3 were lower in si-RNA2 and si-RNA3 groups in Huh7 cells.

**Figure S2** Knockdown of SKA3 does not affect HCC cells in the absence of P53. (a) Western blot analysis showed that SKA3 was effectively knockdown in siRNA2 and siRNA3 groups with low expression of P53 in Hep3B. (b) CCK8 and (c) colony-forming assays were used to determine the cell viability of Hep3B cells transferred with SKA3 siRNAs. The number of colonies per well was counted. (d)(e) Transwell and Scratch wound healing assays was performed to determine the cell motility of Hep3B cells transferred with SKA3 siRNAs. (f)(g) Flow cytometry was performed to determine the cell cycle and cell apoptosis in si-SKA3 groups.

**Figure S3** SKA3 inhibited the interaction between CDK2 and p53 in HCC cells. (a)(e) Total lysates from LM3 cells expressing Flag-SKA3 and HA-CDK2 were subjected to IP with HA Ab or Flag Ab, followed by western blotting using the indicated antibodies (Abs). HA-SKA3 and Flag-CDK2 were used as a loading control. (b)(f) Total lysates from LM3 cells expressing SKA3 or CDK2 were subjected to IP with SKA3 Ab or CDK2 Ab, followed by western blotting using the indicated Abs. (c)(g) Total lysates from LM3 cells expressing HA-P53 and Flag-CDK2 in the presence of GST-SKA3 were subjected to IP with Flag Ab or HA Ab, followed by Western blotting using the indicated Abs. (d)(h) Total lysates from LM3 cells expressing HA-P53 and Flag-CDK2 in the presence of siRNA-SKA3 were subjected to IP with Flag Ab or HA Ab, followed by Western blotting using the indicated Abs.
