# Supplementary figures and images for: SKA3 Promotes tumor growth by regulating CDK2/P53 phosphorylation in hepatocellular carcinoma
Source: Cell Death Dis. 2019 Dec 5;10(12):929. doi: 10.1038/s41419-019-2163-3 (PMC6895034; doi:10.1038/s41419-019-2163-3)

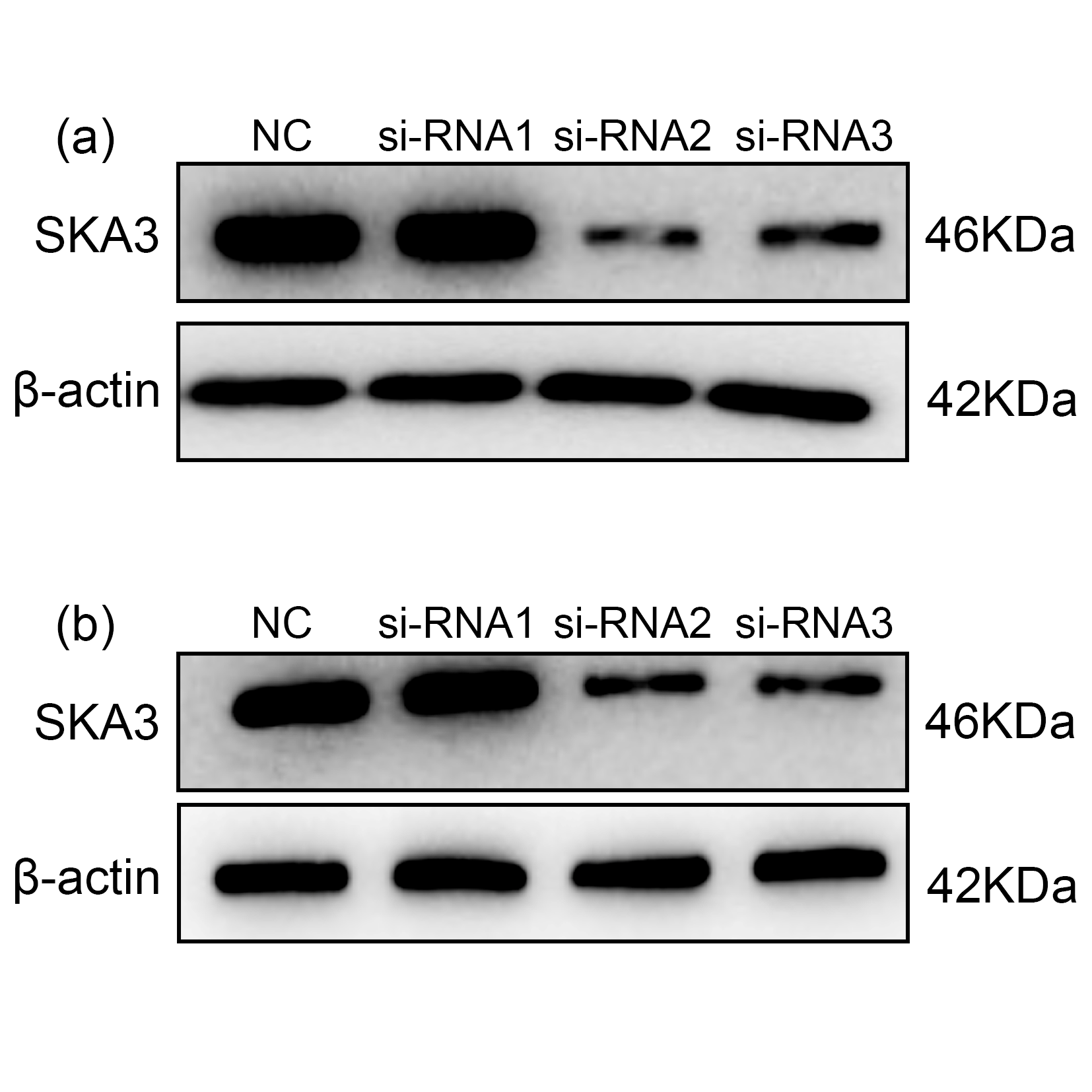

Supplement: Supplementary file 3 — Figure s1 [file 41419_2019_2163_MOESM3_ESM.tif]

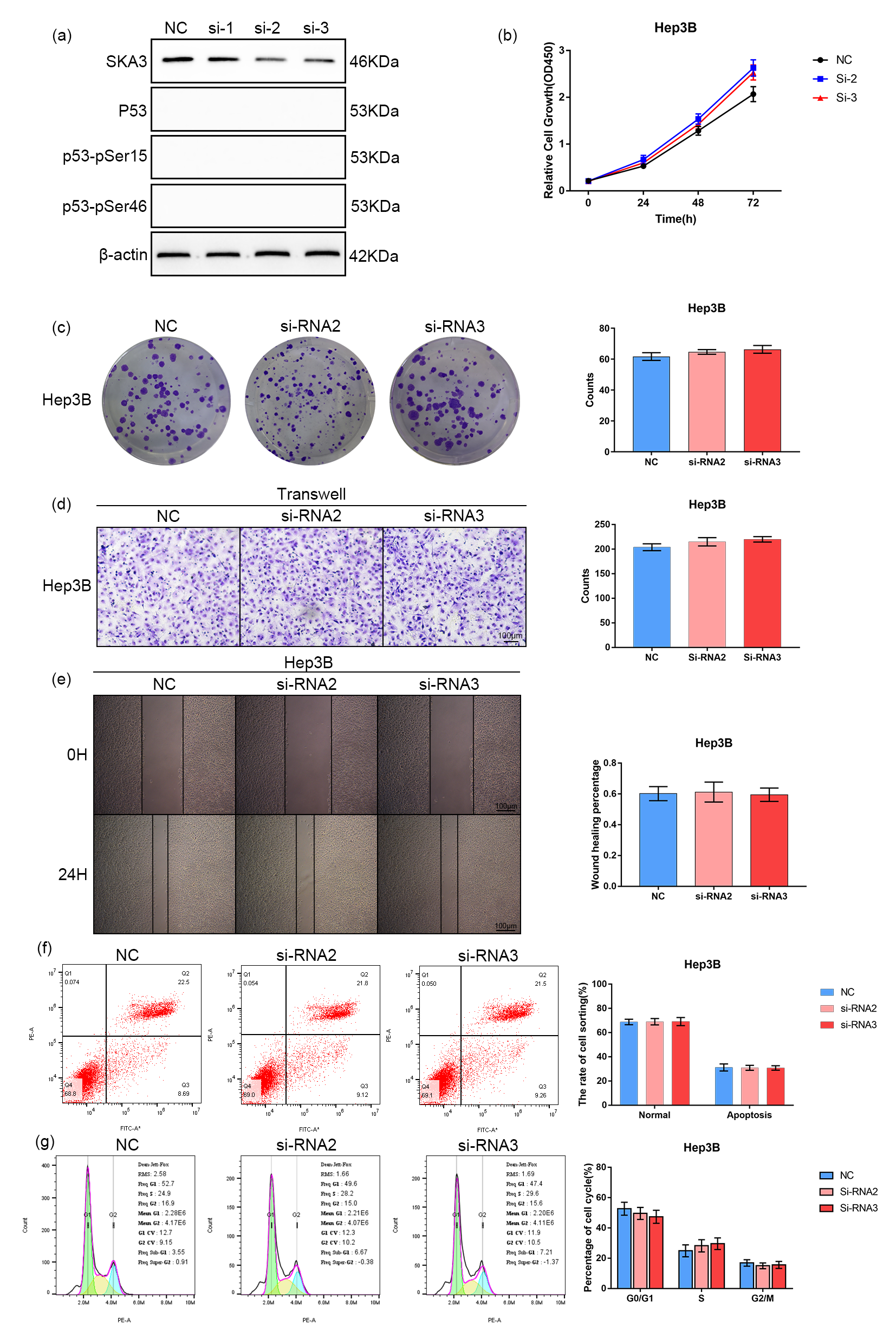

Supplement: Supplementary file 4 — Figure s2 [file 41419_2019_2163_MOESM4_ESM.tif]

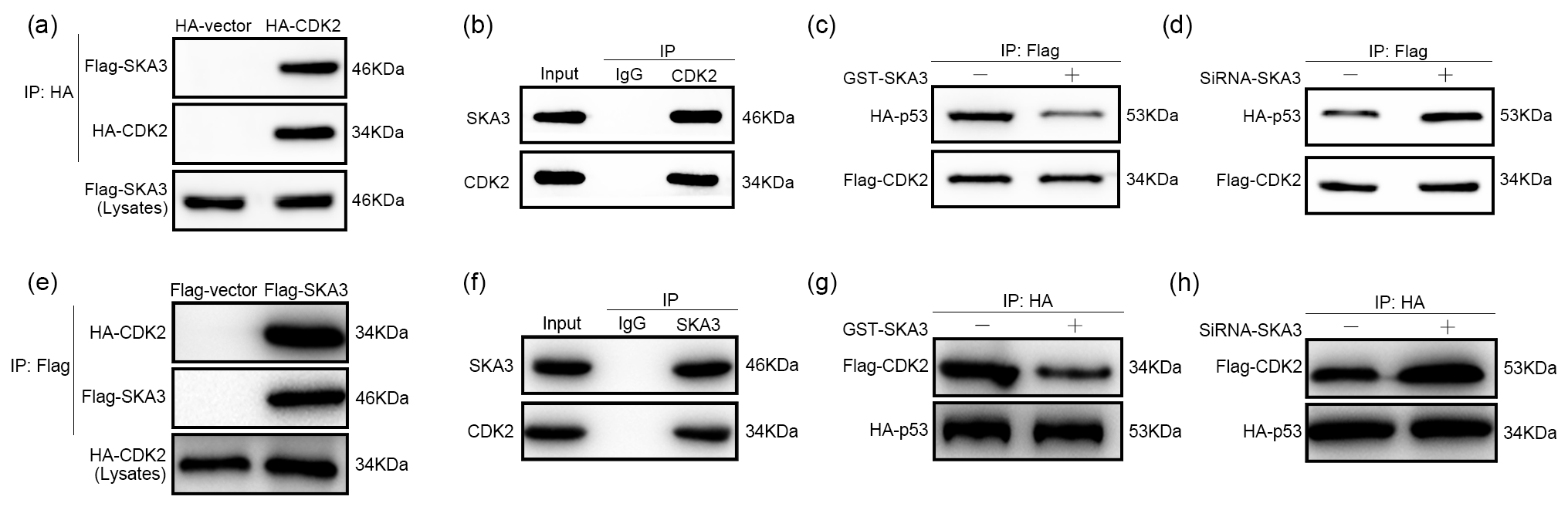

Supplement: Supplementary file 5 — Figure s3 [file 41419_2019_2163_MOESM5_ESM.tif]
